# Prevalence and determinants of goitre among children of South Kordofan state, Sudan, 2021: an urgent need for effective implementation of universal salt iodisation

**DOI:** 10.1017/S1368980023002744

**Published:** 2023-12-14

**Authors:** Azza Elfadil Abdalla, Anfal Mahmoud Altahir, Elfatih A. Hasabo, Salma Salah Alrawa, Amna Mutasim Elazrag, Hayat Abdoallah Ahmed, Hiba Abubakr Ali, Ibrahim Mysara Abdelrazig, Mohamed Yaser Ahmed, Mohamed Alsiddig Alagib, Musab Mohammed Siddig, Rofida Salah Asmally, Salma Mohamed Mohamedelrasheed, Walaa Abdulgadir Elnaiem, Elfatih Mohammed Malik

**Affiliations:** 1 Khartoum Medical Student’s Association, Faculty of Medicine, University of Khartoum, Khartoum, Sudan; 2 CORRIB Research Centre for Advanced Imaging and Core Laboratory, Clinical Science Institute, University of Galway, Galway, Ireland; 3 Discipline of Cardiology, Saolta Healthcare Group, Health Service Executive, Galway University Hospital, Galway, Ireland; 4 Community Medicine Department, Faculty of Medicine, University of Khartoum, Khartoum, Sudan

**Keywords:** Goitre, iodine deficiency disorders, Sudan, universal salt iodisation

## Abstract

**Objective:**

This study aimed to determine the prevalence and determinants of goitre among children aged 6–12 years at South Kordofan state.

**Design:**

This was a cross-sectional facility-based study.

**Setting:**

The study was conducted in twenty villages of South Kordofan state during a medical mission.

**Participants:**

All 575 school-age children (6–12 years) who attended the medical day were examined for clinical assessment of goitre.

**Results:**

The prevalence of goitre among children of South Kordofan was 42·8 % (grade 1: 15·7 %, grade 2: 27·1 %). Only 24·2 % of caregivers reported using iodised salt. Mothers working as farmers (OR: 3·209, CI 95 % 1·437, 7·167; *P* = 0·004) and children of Darforian tribes (OR: 21·799, CI 95 % 2·566, 185·226; *P* = 0·005) were found to be significantly associated with higher prevalence of goitre among children. This contrasts with children of African tribes, where they were found to have less goitre prevalence (OR: 0·432, CI 95 % 0·213, 0·875; *P* = 0·02). Iodised salt utilisation (OR = 0·523, CI 95 % 0·320, 0·854; *P* = 0·01) was found associated with a lower prevalence of goitre.

**Conclusion:**

Even though National Iodine Deficiency Disorders control programs were initiated in Sudan more than 25 years ago, the prevalence of goitre among children in South Kordofan state was alarming (42·8 %). Efforts to improve access to iodised salt, increase utilisation and raise awareness are urgently needed.

Total goitre rate serves as an indicator for assessing iodine deficiency in a community, as it is considered its most common manifestation^(1)^.

Palpation and thyroid ultrasonography is the key means to detect thyroid goitre, with palpation being a time-honored method recommended by the WHO for both the identification and classification of goitre^(1)^.

In the African region, iodine deficiency is considered one of the major health burdens that affect one-third of children^(2)^. On a national level, it is estimated that every year 200 000 Sudanese children are at risk of iodine deficiency^(3)^. Less than 10 % of the population have access to iodised salt^(4)^, which contributed to ranking Sudan as the second most affected with insufficient iodine intake among school-age children in Africa^(2)^.

Sudan’s journey with the goitre began before independence with the first official report in 1952^(5)^. The prevention efforts started with the distribution of iodised oil capsules and passed through the adoption of a universal salt iodisation strategy, issuing several decrees mandating salt fortification, prohibiting the selling of un-iodised salt in several states and setting plans to increase the population’s level of knowledge regarding the disease^(6,7)^.

Universal salt iodisation was recommended by the WHO and UNICEF joint policy committee in 1994 as a global strategy to eliminate iodine deficiency^(8)^. In the same year, the policy was adopted as a long-term national policy^(6)^. According to a multiple indicator cluster survey in 2000, the percentage of households that utilised iodised salt was 0·6 % in the whole country^(9)^. In 2014, the overall percentage increased to 7·6 % and was 9·1 % in South Kordofan, slightly higher than the country average^(9)^.

The prevalence of goitre was assessed in different states in 1997 and found to be 39·9 % in South Kordofan among various age groups^(10)^. Subsequent studies did not include this area, which was an area of conflict till August 2020, when the comprehensive peace agreement was signed.

In terms of preventive measures, many countries – including Sudan – are adopting the Universal Salt Iodisation (USI) strategy aiming to iodise all salt put in use for both humans and animals^(11,12)^. In addition, Sudan had several attempts to fight against the disease; starting with the National Iodine Deficiency Disorders (IDD) control program which managed to introduce the use of iodised oil capsules in highly endemic states. Later, in 2012, plans were set to increase the population’s knowledge regarding the disease as well as increase coverage of iodised salt to 90 % of the population^(13)^.

Five decades of varied efforts directed towards solving the problem of IDD necessitates evaluation, especially in conflict areas such as South Kordofan.

According to WHO^(1)^, schoolchildren are the most accessible segment of the population. Thereby, this study aimed to determine the prevalence of goitre among school-age children in South Kordofan state, in addition to assessing various factors associated with its prevalence.

## Methodology

### Study design and area

The study was conducted from 16 to 20 August 2021 as a part of a medical mission arranged by the Khartoum Medical Students’ Association. The mission provided health services in the form of clinical consultation, free drug prescriptions, health education and epidemiological surveys.

This was a cross-sectional study carried out in South Kordofan state. It is a southern state in the Republic of Sudan. This state has a rich savannah landscape with fertile Nuba Hills covering 50 000 km^2^ of its area. It consists of six localities that contain 1161 villages. The estimated total population of this state is 2 500 000 people, and Kaduqli is the capital of the state.

According to the WHO state report, South Kordofan state has a shortage of health facilities, with only 196 health facilities covering 19 % of the total number of villages in the state. The scarcity of improved drinking water was noted in most villages. Farming and livestock rearing were the main financial resources. The diet is enriched by collecting wild fruits and hunting^(1)^.

### Study population

We targeted 6–12-year-old children residing in the South Kordofan state who attended the medical days since schools were closed at the time of data collection. Additionally, as South Kordofan state is a war area, we were advised by the military not to leave the medical convoy centre during the medical day. We excluded children who were severely ill and could not participate in the study.

### Data collection and sampling

Twenty villages were purposively selected for the study. The selection was based on the need for health services besides relative safety. During each medical day, a convenient sampling approach was used to select the targeted children.

Recruitment of the population started 2 months earlier to the medical mission. Delegation members contacted the village leaders in person regarding the announcement of medical campaigns. Announcements were conducted in public gatherings (mosques, local markets, etc.)

The required sample size was calculated to be 365 using the population proportion formula n = z^2^ P(1 − P)/ d^2^ with the following assumptions: Goitre prevalence in Kordofan zone was found to be 39·1 % (P) as reported by the FAO ‘National country profile of the republic of Sudan’^(14)^, a margin of error (d) of 5 % and CI of 95 %. The sample size was then inflated expecting a 20 % non-response rate. The required sample size was 461.

### Questionnaire and instruments

A structured close-ended interviewer-administered questionnaire was used in this study. It was formed first in the English language and then translated into the Arabic language by bilingual students who were fluent in English and had Arabic as their mother tongue. Medical students responsible for the data collection were trained in interviewing techniques for 3 d before the start of the data collection. Women who were majorly involved in the food preparation of the household were selected as respondents.

The questionnaire consisted of three parts. First part: Socio-demographic data (child’s age, child’s gender, children’s schooling, father’s work, mother’s work, father’s education, mother’s education, tribes’ classification and family size). Second part: Assessment of the mother’s awareness, utilisation and practice towards iodised salt. This section was adapted from a previous study^(15)^. Third part: Assessment of possible predictors of goitre among children such as family history, drinking water source and dietary goitrogens consumption.

Goitrogenic food intake assessment was obtained in the form of frequencies (once per day, 1–3 times per week, once per month, rarely and never). The studied goitrogenic items were collected from previous literature^(16–18)^, and it was selected according to what was included in the diet of South Kordofan state population.

### Assessment of goitre

Physical examination was done for all included children to determine the status of goitre (clinical assessment for goitre) according to the WHO classification criteria^(1)^. Participants were screened for goitre by trained graduates, and ambiguous cases were referred to senior surgical registrars.

The grades for goitre were 0, 1 and 2 where grade 0: neither palpable nor visible goitre, grade 1: palpable goitre but not visible and grade 2: there is a swelling in the neck that is clearly visible.

### Data management and analysis

The data was collected using Kobocollect which is an application used for both online and offline data collection in smartphones, as it is easy to use in rural areas where the internet connection is not available. The students filled out the forms in the offline format and uploaded the responses in the city at the end of each medical day. Data then were entered into a Microsoft Excel database and analysed using Statistical Package for Social Sciences version 25. Descriptive statistics were reported for continuous variables as mean (s d) after checking the Normality of the distribution of continuous variables using the Kolmogorov–Smirnov test (cut-off at *P* = 0·05). Categorical data were described using frequencies and percentages [n (%)]. The *χ* ^2^ test and Fisher’s exact test were used to find if there were any significant differences between groups with and without goitre. A multivariate binary logistic regression was used to find factors associated with goitre.

## Results

### Socio-demographic characteristics

A total of 575 schoolchildren between 6 and 12 years old were included in the study. There were no refusals for participation by caregivers. Of these children, 350 (60·9 %) were females and 225 (39·1 %) were males, resulting in an overall 1·56: 1 female to male ratio. Most of the participants (*n* 234, 50·4 %) were Nubians. The most common age among participants was 7 years old (20·5 %), and the mean family size was 7·85 (4·2 sd). The majority of children (*n* 377, 65·6 %) were primary school pupils. Parental educational level was mostly primary; (44·5 %) among mothers and (36·2 %) among fathers. Most of the fathers were government employees (*n* 178, 31·0 %), farmers (*n* 145, 25·2 %) or daily laborers (*n* 119, 20·7 %). The majority of mothers (*n* 379, 65·9 %) were housewives. Upon comparing the mother’s work and tribe with goitre status through binary analysis, a statistically significant difference was noted (*P* < 0·001) (Table 1).

**Table 1:** Socio-demographic characteristics of participating children and their parents, South Kordofan, 2021

Characteristic	*n*			Presence of goitre				*P*-value*
		Overall, *n* 575†		No, *n* 329†		Yes, *n* 246†		
		*n*	%	*n*	%	*n*	%	
Age, years	575							>0·9
6		92	16·0	56	17·0	36	14·6	
7		118	20·5	67	20·4	51	20·7	
8		108	18·8	59	17·9	49	19·9	
9		61	10·6	38	11·6	23	9·3	
10		94	16·3	53	16·1	41	16·7	
11		45	7·8	23	7·0	22	8·9	
12		57	9·9	33	10·0	24	9·8	
Gender	575							0·11
Male		225	39·1	138	41·9	87	35·4	
Female		350	60·9	191	58·1	159	64·6	
Child schooling	575							0·5
Does not go to school		76	13·3	45	13·7	31	12·7	
Informal		2	0·3	1	0·3	1	0·4	
Pre-school		118	20·6	74	22·5	44	18·0	
Primary school		377	65·8	209	63·5	168	68·9	
Father’s work	571							0·18
Day laborer		119	20·8	72	22·0	47	19·3	
Farmer		145	25·4	75	22·9	70	28·7	
Governmental employee		178	31·2	94	28·7	84	34·4	
Merchant		15	2·6	9	2·8	6	2·5	
Private employee		17	3·0	11	3·4	6	2·5	
Shepherd		4	0·7	1	0·3	3	1·2	
Not working		63	11·0	44	13·5	19	7·8	
Others		30	5·3	21	6·4	9	3·7	
Mother’s work	573							**<0·001**
Day laborer		55	9·6	38	11·6	17	6·9	
Farmer		117	20·4	45	13·8	72	29·3	
Governmental employee		9	1·6	4	1·2	5	2·0	
Merchant		6	1·0	3	0·9	3	1·2	
Private employee		3	0·5	3	0·9	0	0·0	
Housewife		379	66·1	233	71·3	146	59·3	
Others		4	0·7	1	0·3	3	1·2	
Father’s education	555							0·7
Illiterate		185	33·3	110	34·6	75	31·6	
Informal		18	3·2	8	2·5	10	4·2	
Primary school		208	37·5	119	37·4	89	37·6	
Secondary school		114	20·5	65	20·4	49	20·7	
University		29	5·2	16	5·0	13	5·5	
Postgraduate		1	0·2	0	0·0	1	0·4	
Mother’s education	572							0·065
Illiterate		238	41·6	152	46·5	86	35·1	
Informal		11	1·9	5	1·5	6	2·4	
Primary school		256	44·8	138	42·2	118	48·2	
Secondary school		58	10·1	28	8·6	30	12·2	
University		9	1·6	4	1·2	5	2·0	
Tribes’ classification	464							**<0·001**
Nuba		234	50·4	126	48·3	108	53·2	
Arab		151	32·5	84	32·2	67	33·0	
African		64	13·8	50	19·2	14	6·9	
Darforian		15	3·2	1	0·4	14	6·9	
Family size								
Mean	573	7·85		8·12		7·48		0·072
sd		4·2		4·54		3·67		

* Pearson’s *χ* ^2^ test; Fisher’s exact test; two sample *t* test.

† *n* (%); Mean (sd).

Boldface was used for the statistically significant *P*-values.

### Goitre prevalence

The overall goitre prevalence in all villages was (42·8 %). Regarding the WHO grading, grade 2 goitre prevalence was higher than grade 1, making 27·1 % *v*. 15·7 %, respectively. Some villages had a prevalence exceeding half of the population, and these are Samasem, Sharaf Aldai, Alsema East, Albardab, Tello, Engmina and Alshaaeer Almatar (see online supplementary material, Supplemental Table 1).

### Caregiver’s knowledge, attitude and utilisation of iodised salt

Of the 575 interviewed caregivers, only 303 (52·7 %) heard of iodised salt, and a mere 139 (24·2 %) used it. When they were asked about natural sources of iodine, the majority (*n* 510, 88·7 %) stated that they did not know. Those who used iodised salt showed favourable practice, with 138 (91·3 %) of them keeping it in closed containers. Most of them (*n* 123, 89·1 %) stored it for less than two months, and (*n* 116, 84·1 %) of them kept it away from fire and sunlight. Regarding the addition of salt when cooking, only 46 (33·3 %) used it appropriately at the end of cooking (Table 3).

**Table 2:** Family history of thyroid problems and sources of drinking water among participating children, South Kordofan, 2021

Characteristic	*n*			Presence of goitre				*P*-value*
		Overall, *n* 575†		No, *n* 329†		Yes, *n* 246†		
		*n*	%	*n*	%	*n*	%	
Did any of your family suffer from a thyroid problem?	575							0·8
No		338	58·8	197	59·9	141	57·3	
Yes		235	40·9	131	39·8	104	42·3	
I do not know		2	0·3	1	0·3	1	0·4	
If yes what was the degree of relativeness	234							0·4
First degree		71	30·3	36	27·5	35	34·0	
Second degree		144	61·5	82	62·6	62	60·2	
Third degree		7	3·0	6	4·6	1	1·0	
Far		12	5·1	7	5·3	5	4·9	
Sources of drinking water:	572							0·2
Governmental water supply network		40	7·0	25	7·6	15	6·1	
Underground water		440	76·9	257	78·6	183	74·7	
Surface water		82	14·3	42	12·8	40	16·3	
Rain		10	1·7	3	0·9	7	2·9	

* Pearson’s *χ* ^2^ test; Fisher’s exact test; two sample *t* test.

† *n* (%); Mean (sd).

**Table 3:** Knowledge, attitude and utilisation of iodised salt among caregivers, South Kordofan, 2021

Characteristic	*n*			Presence of goitre				*P*-value*
		Overall, *n* 575†		No, *n* 329†		Yes, *n* 246†		
		*n*	%	*n*	%	*n*	%	
What are the most important natural sources of iodine?	575							
Seafood	575	51	8·9	29	8·8	22	8·9	>0·9
Egg	575	6	1·0	4	1·2	2	0·8	>0·9
Meet	575	5	0·9	3	0·9	2	0·8	>0·9
Milk and milk products	575	5	0·9	2	0·6	3	1·2	0·7
Fruits and vegetables	575	13	2·3	8	2·4	5	2·0	0·8
I do not know	575	510	88·7	292	88·8	218	88·6	>0·9
Other	575	1	0·2	0	0·0	1	0·4	0·4
Have you heard about iodised salt? (Yes)	575	303	52·7	161	48·9	142	57·7	**0·037**
What type of salt do you use?	575							**0·004**
Iodised		139	24·2	86	26·1	53	21·5	
Regular		364	63·3	191	58·1	173	70·3	
I do not know		72	12·5	52	15·8	20	8·1	
Where do you store iodised salt?	138							0·12
Closed container		126	91·3	81	94·2	45	86·5	
Open container		12	8·7	5	5·8	7	13·5	
Duration of household iodised salt storage	138							**0·014**
Less than two months		123	89·1	81	94·2	42	80·8	
More than two months		15	10·9	5	5·8	10	19·2	
Do you expose it to sunlight?	138							0·5
Yes		22	15·9	15	17·6	7	13·2	
No		116	84·1	70	82·4	46	86·8	
Do you store it near the fire?	139							0·14
Yes		21	15·1	16	18·6	5	9·4	
No		118	84·9	70	81·4	48	90·6	
When do you add it?	138							0·2
Beginning		66	47·8	46	54·1	20	37·7	
Middle		26	18·8	14	16·5	12	22·6	
Ending		46	33·3	25	29·4	21	39·6	

* Pearson’s *χ* ^2^ test; Fisher’s exact test.

† *n* (%).

Boldface was used for the statistically significant *P*-values.

There were significant statistical differences in the comparison of previous knowledge about iodised salt (*P* = 0·037), type of utilised salt (*P* = 0·004) and duration of storage (*P* = 0·014) with the presence of goitre (Table 3).

### Goitrogens and drinking water source of participants

Investigating goitrogen consumption by children revealed that sorghum (61·6 %) and onion (91·1 %) were used once daily by most of the participants. However, both were not significantly associated with the presence of goitre. In contrast, maize and sweet potato consumption showed significant differences (Table 4).

**Table 4:** Frequency of dietary goitrogens consumption among participating children, South Kordofan, 2021

Characteristic	*n*			Presence of goitre				*P*-value*
		Overall, *n* 575†		No, *n* 329†		Yes, *n* 246†		
		*n*	%	*n*	%	*n*	%	
Dietary goitrogens								
Sorghum	575							0·3
Once per day		354	61·6	198	60·2	156	63·4	
1–3 times per week		189	32·9	107	32·5	82	33·3	
Once per month		16	2·8	13	4·0	3	1·2	
Rarely		11	1·9	7	2·1	4	1·6	
Never		5	0·9	4	1·2	1	0·4	
Millet	575							0·2
Once per day		11	1·9	5	1·5	6	2·4	
1–3 times per week		80	13·9	40	12·2	40	16·3	
Once per month		64	11·1	41	12·5	23	9·3	
Rarely		176	30·6	94	28·6	82	33·3	
Never		244	42·4	149	45·3	95	38·6	
Cassava	575							0·8
Once per day		5	0·9	3	0·9	2	0·8	
1–3 times per week		24	4·2	12	3·6	12	4·9	
Once per month		25	4·3	14	4·3	11	4·5	
Rarely		56	9·7	36	10·9	20	8·1	
Never		465	80·9	264	80·2	201	81·7	
Onion	575							0·05
Once per day		524	91·1	290	88·1	234	95·1	
1–3 times per week		36	6·3	28	8·5	8	3·3	
Once per month		4	0·7	3	0·9	1	0·4	
Rarely		5	0·9	4	1·2	1	0·4	
Never		6	1·0	4	1·2	2	0·8	
Maize	575							**0·021**
Once per day		108	18·8	57	17·3	51	20·7	
1–3 times per week		103	17·9	61	18·5	42	17·1	
Once per month		39	6·8	27	8·2	12	4·9	
Rarely		297	51·7	161	48·9	136	55·3	
Never		28	4·9	23	7·0	5	2·0	
Sweet potato	575							**<0·001**
Once per day		7	1·2	2	0·6	5	2·0	
1–3 times per week		285	49·6	141	42·9	144	58·5	
Once per month		107	18·6	74	22·5	33	13·4	
Rarely		101	17·6	67	20·4	34	13·8	
Never		75	13·0	45	13·7	30	12·2	
Lima beans	575							0·5
Once per day		9	1·6	5	1·5	4	1·6	
1–3 times per week		195	33·9	105	31·9	90	36·6	
Once per month		117	20·3	64	19·5	53	21·5	
Rarely		115	20·0	67	20·4	48	19·5	
Never		139	24·2	88	26·7	51	20·7	
Pumpkin	575							0·5
Once per day		14	2·4	8	2·4	6	2·4	
1–3 times per week		58	10·1	31	9·4	27	11·0	
Once per month		36	6·3	24	7·3	12	4·9	
Rarely		375	65·2	208	63·2	167	67·9	
Never		92	16·0	58	17·6	34	13·8	

* Pearson’s *χ* ^2^ test; Fisher’s exact test.

† *n* (%).

Boldface was used for the statistically significant *P*-values.

The major sources of drinking water were underground water which was used by (*n* 440, 76·9 %) of participants followed by surface water (*n* 82, 14·3 %). Governmental water supply networks and rain were used by (*n* 40, 7·0 %) and (*n* 10, 1·7 %), respectively. There was no significant difference between sources of water when compared with the presence of goitre (*P* = 0·2) (Table 2).

### Family history of thyroid disease

A number of 235 (40·9 %) participants had a family member with thyroid disease. The relation was a first degree in 71 (30·3 %) of them. Family history did not show a statistically significant difference when compared with the presence of goitre (*P* = 0·8) (Table 2).

### Predictors of goitre

Only variables that showed significance in the binary analysis were entered in the multivariate logistic regression model. Regarding predictors of goitre, children with mothers working as farmers (OR: 3·209, CI 95 % 1·437, 7·167; *P* = 0·004) and children of Darforian tribes (OR: 21·799, CI 95 % 2·566, 185·226; *P* = 0·005) were found to be significantly associated with higher prevalence of goitre among children. This is in contrast to children of African tribes, where they were found to have less goitre prevalence (OR: 0·432, CI 95 % 0·213, 0·875; *P* = 0·02). Another factor linked to lower goitre prevalence was the use of iodised salt (OR = 0·523, CI 95 % 0·320, 0·854; *P* = 0·01) (Table 5).

**Table 5:** Multivariate logistic regression showing predictors for presence of goitre

Variables	Multivariate logistic regression		
	OR	95 % CI	*P*-value
		Lower, Upper	
Mothers’ work			
Day laborer (Reference)	–	–	–
Farmer	3·209	1·437, 7·167	**0·004**
Governmental employee	1·645	0·335, 8·062	0·540
Merchant	2·974	0·420, 21·038	0·275
Private employee	0·000	0·000	0·999
House wife	1·392	0·688, 2·816	0·358
Others	3·769	0·279, 50·976	0·318
Tribes classification			
Nuba	–	–	–
Arab	1·134	0·710, 1·813	0·598
African	0·432	0·213, 0·875	**0·020**
Darforian	21·799	2·566, 185·226	**0·005**
Have you heard about iodised salt?			
No (Reference)	–	–, –	–
Yes	1·271	0·813, 1·986	0·292
What type of salt you use?			
Regular (Reference)	–	–	–
Iodised	0·523	0·320, 0·854	**0·010**
I do not know	0·765	0·376, 1·557	0·460
Maize			
Once per day (Reference)			
1–3 times per week	0·986	0·499, 1·948	0·967
Once per month	0·551	0·213, 1·427	0·220
Rarely	1·119	0·634, 1·974	0·699
Never	0·291	0·065, 1·311	0·108
Sweet potato			
Once per day (Reference)	–	–	–
1–3 times per week	0·542	0·081, 3·616	0·527
Once per month	0·185	0·026, 1·292	0·089
Rarely	0·279	0·040, 1·952	0·198
Never	0·537	0·073, 3·972	0·543

Boldface was used for the statistically significant *P*-values.

## Discussion

South Kordofan, a state experiencing unrest and war for a long time, suffers from poor socio-economy, lack of basic health services and high levels of illiteracy leading to malpractices, as what was observed in this medical mission. These factors contributed to the susceptibility of the population to retract pervasive nutritional deficiencies, including IDD.

This study reported a 42·8 % prevalence of goitre among 6–12-year-old children of South Kordofan state, which is classified as severe iodine deficiency^(19)^.

A high prevalence was also noted in other western states^(20)^, reporting 87 %, 66 % and 64·1 %, in Darfur, Elfashir and Nyala, respectively. In contrast, Khartoum^(20)^ and Port Sudan^(21)^ have reported only 14·3 % and 17·1 %, respectively.

There is lateralisation of the prevalence of goitre towards the western states, as was substantiated by this study. This can be explained by distant locations from the center, instability, war and low socio-economic status. Another important factor is ecological; western states have a mountainous and highlands topography, which is exposed to soil erosion, rendering food crops to be iodine deficient. This could be compared with Port Sudan^(21)^, a coastal city with a relatively lower prevalence of goitre (17·1 %). The study^(21)^ also detected a high urinary iodine concentration among children in the city, which reported that it could be due to the consumption of over-iodised salt.

South Kordofan’s prevalence of goitre among children largely exceeded other developing countries, such as Ethiopia (39·9 %)^(15)^, Niger (13·2 %)^(22)^ and India (23·6 %)^(23)^. This may be attributed to the successful implementation of the IDD elimination program in these countries.

In 1972, the government of Sudan concluded that iodine deficiency is the main risk factor associated with goitre in the country^(5)^. Several IDD control programs were established in Sudan since then, but a study conducted in 2010^(20)^ covering nine states reported a goitre prevalence of 38·8 %, affirming that no significant improvement in the population’s iodine status has occurred.

Reports indicate that no progress ensued in the execution of these programmes, and most of them have dismissed their activity. The last Multiple Indicator Cluster Survey^(9)^, stated that Sudan does not have a regulating law for salt iodisation. A UNICEF report in the year 2005^(24)^ showed that only 1 % of households in Sudan had accessibility to iodised salt, which was then increased to 7·6 % as reported by Multiple Indicator Cluster Survey in 2014^(9)^.

USI implementation is proven to be the most efficient method of reduction of IDD^(25)^. For instance, China succeeded in obtaining a 25·2 % decrease in total goitre rate after the execution of USI^(26)^. However, in Sudan, USI has not been established well in all states. The results of this study show that only 52·7 % of the participants heard about iodised salt, and only 24·2 % are using it. Iodised salt utilisation was found to be protective against goitre (OR = 0·501, CI 95 % 0·303, 0·830; *P* = 0·007). A national study reported that only 8 % of the South Kordofan population had access to iodised salt, and only 3·6 % of the salt at local markets is adequately iodised^(27)^. According to their results, urban populations are more likely to use iodised salt. Even in the Sudanese metropolis, only 27·9 % of students in Bahri city reported iodised salt utilisation by their households^(28)^. These low figures emphasise the defect in the supply chain of adequately iodised salt to the centre and periphery of the country, which reflects the defects in the federal system upon implementing USI. Programme priority must be based on situation analysis of USI in each state, offering more efficient autonomous governance of the programme^(27)^.

Problems facing USI implementation in Sudan may be attributable to the low rate of production and use of iodised salt in the country as issued in the Ministerial declaration (2003)^(21)^, which called for the obligatory iodination of all salt that has subsequently failed to be applied.

An additional factor contributing to the impediment of USI implementation is the possibility of inadequate iodisation of salt, related to factors such as inaccessibility, storage problems and inappropriate practices. For instance, shown in the case of Ethiopia, has succeeded to reach 89 % of salt being iodised in the country^(29)^, with only 32·5 % of the household salts being adequately iodised^(15)^. Similarly, India has shown to have 22 % of households consume salt with inadequate iodine^(30)^.

Most of our study participants reported good practice regarding salt storage, with 91 % storing it in closed containers, and about 84 % not exposing it either to sunlight or fire. Moreover, 89·1 % of participants stored their salt for less than 2 months (*P* < 0·05) (Table 3). Even though 21·5 % of those who present goitre use iodised salt, inadequate cooking of the salt could be the suggested cause.

All these inputs reflect that USI in Sudan is a public policy issue, rather than an individual-level problem.

In this study, we found that the mothers’ occupation is significantly associated with the presence of goitre among their children, while the father’s occupation shows no significant differences. Children of farmer mothers are more likely to have goitre (OR: 2·984, CI 95 % 1·320, 6·747; *P* = 0·009). This contrasts with an Ethiopian study^(15)^, where children of housewives were found to have more goitre compared with working mothers. This might be related to the effortful job of farming and the possible burn-out-driven neglect. Children of farmer mothers are more likely to consume raw goitrogens in higher frequencies, which may explain why they are more likely to have goitre. Due to the nature of the mother’s job, they are more likely to live in poverty, suffer neglect and consume non-iodised more than iodised salt.

Iodine can be acquired from drinking water and other beverages, but food represents the most iodine intake^(31)^. Similar studies among Sudanese children in Port Sudan and Jabal Awliya areas had recorded a lower prevalence of goitre compared with our results (17·1 % and 14 %, respectively), which may be predominantly caused by the difference in dietary habits, as these areas have fish contributing largely to their diet, while it is not widespread in South Kordofan^(19)^.

Despite the high consumption rate of onion in the area (91 %), the analysis does not show any significant association with goitre, which is opposite to a study conducted in the River Nile State^(32)^, which is mostly inhabited by Arab tribes. This may be due to a genetic effect among different ethnicities that forces its goitrogenic effect.

Soil and water iodine content is directly related to the iodine content of food^(33)^. Seawater and groundwater retain more iodine concentration than surface water sources^(23)^.

However, the source of water was not significantly associated with goitre in this study. In contrast to an Indian study, where the prevalence of goitre was significantly higher in children who used surface water sources for drinking purposes compared with those who used groundwater^(34)^. Coastal areas are known to contain more iodine in water compared with mountainous areas as the iodine-rich layer of soil is cleared by heavy rains and replaced by iodine-poor crystalline rocks^(35)^. This fact may suggest the possibility that all water sources in this study are iodine deficient, making no difference upon comparison. The iodine content of the water should be analysed. Worth noting that in Elfigaiga area in River Nile State in Sudan, the water supply was not found to be significant, and upon investigation, they found all sources to be iodine sufficient^(32)^.

Contaminated water was found to be associated with goitre in some studies^(36,37)^. Safe Water Supply, Sanitation, and Hygiene is a project that accompanied this medical mission. Microbiological analysis of the main sources of water in the study area was done and revealed that 100 % of surface water, and 39·4 % of groundwater were contaminated by *E. coli*. The report also showed that 47·6 % of household samples were also contaminated. This may explain the high prevalence of goitre since *E. coli* and organic material bind the iodine in water, rendering it iodine deficient^(38)^.

Family history has not shown a significant association with goitre. In contrast to studies in Denmark^(39)^and India^(40)^, where the familial occurrence of goitre was associated with goitre prevalence. The nutritional nature of goitre in South Kordofan may mask familial effects. Although our results showed that Darforian tribes are more likely to develop goitre in contrast to Arab and Nuba, the effect of genetics among Africans is argued to play a less important role than environmental factors, such as poor socio-economy and nutrition^(41)^.

This study is not without limitations. First, the inherent cross-sectional study design nature does not allow us to make conclusions about causalities. Second, the non-probability sampling we used put the results’ generalisability under question. Third, logistic and financial support shortages were hurdled by measuring important relevant parameters of iodine status in the population, such as urinary iodine concentration, iodine concentration in drinking water and households’ salt, as well as ultrasonography. Moreover, recall bias was prevailing in dietary parameters reported in this study.

Despite these limitations, this study reported the first goitre statistics in South Kordofan among the paediatric population. Moreover, it had a large sample size and is the first study in Sudan to report a variety of determinants of goitre.

The USI’s effective implementation and consistent evaluation are crucial to eliminating this endemic public health issue. Increasing the availability of iodised salt by upsurging manufacturing and equal distribution to all vulnerable populations should be given priority in the implementation steps. The evident illiteracy and lack of awareness about iodised salt in this study mandate necessary educational interventions in all governmental and non-governmental institutions.

### Conclusion

Even though IDD control programs were initiated in Sudan more than 25 years ago, the prevalence of goitre in South Kordofan state among children in this study was found alarming (42·8 %). Efforts to improve access to iodised salt, increase utilisation and raise awareness are urgently needed. The State Ministry of Health together with civil societies could play a vital role in this movement.

## Supporting information

Abdalla et al. supplementary material Abdalla et al. supplementary material

## Supplementary material

For supplementary material accompanying this paper visit https://doi.org/10.1017/S1368980023002744
