# Supplementary material for: Prevalence and determinants of goitre among children of South Kordofan state, Sudan, 2021: an urgent need for effective implementation of universal salt iodisation
Source: Public Health Nutr. 2023 Dec 14;27(1):e18. doi: 10.1017/S1368980023002744 (PMC10830365; doi:10.1017/S1368980023002744)
Supplement: Abdalla et al. supplementary material [file S1368980023002744sup001.docx]

| **Supplementary table 1:** Prevalence of goiter among school children in targeted twenty villages of Kadogli, South Kordofan state | | | | | |
| --- | --- | --- | --- | --- | --- |
| **Villages** | **Names of villages** | | | | |
| **Group A villages** | **A1**, N = 39*^1^* | **A2**, N = 26*^1^* | **A3**, N = 44*^1^* | **A4**, N = 32*^1^* | **A5**, N = 12*^1^* |
| **Presence of goiter (Yes)** | 17 (43.6%) | 17 (65.4%) | 31 (70.5%) | 24 (75.0%) | 6 (50.0%) |
| **Level of goiter** |  |  |  |  |  |
| Grade 0 | 22 (56.4%) | 9 (34.6%) | 13 (29.5%) | 8 (25.0%) | 6 (50.0%) |
| Grade 1 | 6 (15.4%) | 4 (15.4%) | 10 (22.7%) | 6 (18.8%) | 2 (16.7%) |
| Grade 2 | 11 (28.2%) | 13 (50.0%) | 21 (47.7%) | 18 (56.2%) | 4 (33.3%) |
| **Group B villages** | **B1**, N = 27*^1^* | **B2**, N = 32*^1^* | **B3**, N = 31*^1^* | **B4**, N = 22*^1^* | **B5**, N = 40*^1^* |
| **Presence of goiter (Yes)** | 3 (11.1%) | 11 (34.4%) | 9 (29.0%) | 6 (27.3%) | 10 (25.0%) |
| **Level of goiter** |  |  |  |  |  |
| Grade 0 | 24 (88.9%) | 21 (65.6%) | 22 (71.0%) | 16 (72.7%) | 30 (75.0%) |
| Grade 1 | 3 (11.1%) | 7 (21.9%) | 7 (22.6%) | 4 (18.2%) | 5 (12.5%) |
| Grade 2 | 0 (0.0%) | 4 (12.5%) | 2 (6.5%) | 2 (9.1%) | 5 (12.5%) |
| **Group C villages** | **C1**, N = 36*^1^* | **C2**, N = 23*^1^* | **C3**, N = 23*^1^* | **C4**, N = 27*^1^* | **C5**, N = 18*^1^* |
| **Presence of goiter (Yes)** | 3 (8.3%) | 14 (60.9%) | 3 (13.0%) | 11 (40.7%) | 15 (83.3%) |
| **Level of goiter** |  |  |  |  |  |
| Grade 0 | 33 (91.7%) | 9 (39.1%) | 20 (87.0%) | 16 (59.3%) | 3 (16.7%) |
| Grade 1 | 0 (0.0%) | 0 (0.0%) | 2 (8.7%) | 7 (25.9%) | 9 (50.0%) |
| Grade 2 | 3 (8.3%) | 14 (60.9%) | 1 (4.3%) | 4 (14.8%) | 6 (33.3%) |
| **Group E villages** | **E1**, N = 34*^1^* | **E2**, N = 33*^1^* | **E3**, N = 22*^1^* | **E4**, N = 21*^1^* | **E5**, N = 33*^1^* |
| **Presence of goiter (Yes)** | 3 (8.8%) | 18 (54.5%) | 17 (77.3%) | 16 (76.2%) | 12 (36.4%) |
| **Level of goiter** |  |  |  |  |  |
| Grade 0 | 31 (91.2%) | 15 (45.5%) | 5 (22.7%) | 5 (23.8%) | 21 (63.6%) |
| Grade 1 | 2 (5.9%) | 7 (21.2%) | 4 (18.2%) | 3 (14.3%) | 2 (6.1%) |
| Grade 2 | 1 (2.9%) | 11 (33.3%) | 13 (59.1%) | 13 (61.9%) | 10 (30.3%) |
| *^1^*n (%) | | | | | |
